# Supplementary material for: A model of long-term population growth with an application to Central West Argentina
Source: PLoS One. 2024 Aug 7;19(8):e0307703. doi: 10.1371/journal.pone.0307703 (PMC11305588; doi:10.1371/journal.pone.0307703)
Supplement: S1 Appendix — (PDF) [file pone.0307703.s001.pdf]

Supporting Information Appendix for “A model of long-term population growth with an application to Central West Argentina”

Jacob Freeman[1,2*◑], Adolfo F. Gil[3,4◑], Eva A. Peralta[3◑], Fernando Franchetti[3◑], José Manuel López[5◑], Gustavo Neme [3◑]

1 Anthropology Program Utah State University, Logan, UT USA
2 The Ecology Center, Utah State University, Logan, UT USA
3 Instituto de Evolución, Ecología Histórica y Ambiente, Consejo Nacional de Investigaciones Científicas y Técnicas (IDEVEA, CONICET & UTN). J. J. Urquiza 314, San Rafael, Mendoza, Argentina
4 Facultad de Filosofía y Letras, Universidad Nacional de Cuyo, Mendoza, Argentina
5 Instituto Argentino de Investigaciones de las Zonas Áridas (IADIZA, CCT CONICET Mendoza), Argentina, 5500.

◑These authors contributed equally to this work.
*jacob.freeman@usu.edu

This document provides supporting analyses for “A model of long-term population growth with an application to Central West Argentina.” The document contains two sections. The first section discusses the possibility that bias related to the preservation of archaeological remains impacts the main results of our archaeological data analysis. The second section discusses the distribution of stable isotopes in human bone among the archaeological areas of Central West Argentina (CWA) in more detail.

Taphonomy

In archaeological research, taphonomy describes the processes by which the byproducts of human activity become preserved as traces of past human systems. One potential concern related to our analysis of archaeological radiocarbon is that the time-period from 4,000 to approximately 2,500 cal BP was really not a period of low population density in each area of CWA. Rather, the increase in dated archaeological remains after approximately 2,950 to 1,900 cal BP may result from taphonomic bias. Here, taphonomic bias refers to the idea that material remains closer to the present are more likely to be preserved than material remains deposited earlier in time. Thus, more material will be available for radiocarbon dating from time periods closer to the present. To investigate this possibility more, we explored two lines of evidence: Material density from a stratified cave deposit in the region, and the effect of using a global taphonomic adjustment. Both lines of evidence suggest that the increase in date frequencies is not simply the result of preservation bias.

First, archaeologists in CWA have long argued that population declined during the Mid-Holocene and did not raise again until the Late Holocene (i.e., after 3,000 cal BP). This argument comes from the distribution of archaeological sites and the analysis of stratified cave deposits. For example, Neme et al. study the change in fauna, lithic, ceramic, and other material frequencies over time in a stratified cave site in the Central area of CWA [1]. After 7,000 Cal BP, material classes decline in frequency per 10 centimeter arbitrary level. However, after 2,100 cal BP material frequencies increase

and then increase again after 1,300 cal BP [1, e.g., Fig. 4]. These patterns suggest that occupational intensity in CWA increased after 2,500 cal BP, as noted in the main paper.

Second, we created Figs. 1-3. These Figs. compare the mean KDE estimates used in the main paper with mean KDE estimates transformed by a global taphonomic adjustment [2–4]. Please keep in mind two important points as we discuss these results.

(1) At this point, we assume that using the global adjustment "corrects" the mean KDEs for the loss of archaeological radiocarbon. This is an assumption that is overly simplistic, but it is only a starting point. (2) To interpret the changes in the mean KDEs illustrated in Figs. 1-3, it is important to understand how the global taphonomic adjustment works. The adjustment procedure is based on an argument from analogy. Researchers collected times-series of non-archaeological dated sediments and remains (like fauna deposits) and then modeled the loss of these records over the last approximately 30,000 years as a function of time [2–4]. The resulting model is a power function,

$$l = 21149.57(t + 1788.03)^{-1.26}. \tag{1}$$

Where l is the loss of dated contexts, t is time, and 21149.57 and -1.26 are parameters that determine the shape of a power function. The analogical argument is that the loss of dated contexts in archaeological sites is the same as in non-archaeological contexts and is described by a highly general function of sediment/context loss. To adjust radiocarbon time-series, the typical procedure is to divide the loss function (a non-linear power function) by the mean KDE (or SPD) of an area to adjust for taphonomic loss. This procedure results in the transformation of the curves observed between graphs A and B in Figs. 1-3

Fig. 1A and B compare the mean KDE and taphonomically adjusted KDE in the Northern area of CWA. There are two key points. (1) The adjusted KDE still displays the long-term period of expansion from 2,950 to approximately 1,700 cal BP noted in the main text. The long-term increase in radiocarbon is the same in both the un-adjusted and taphonomically adjusted mean KDEs. (2) The adjusted KDE makes the population decline after 1,100 cal BP appear much more dramatic than in the un-adjusted KDE. This change would better support our speculation that the Northern area experienced more sustained growth but, as a consequence, a larger population recession. However, we are skeptical of the adjusted KDE, and we suspect that the larger decline after 1,100 cal BP is an artifact of the adjustment procedure (see below).

Fig. 2A and B compare the mean KDE and taphonomically adjusted KDE in the Central area of CWA. As above, there are two key points. (1) The adjustment does not change the observation that the mean KDE suddenly increases at about 2,500 cal BP coincident with the adoption of domesticates. In fact, the taphonomic adjustment enhances this pattern. (2) As above, the adjustment decreases the mean KDE peak around 1,100 cal BP, and creates the impression of a population recession below the long-term carrying capacity of the area after 1,100.

Finally, Fig. 3A and B compare the mean KDE and taphonomically adjusted KDE in the Southern area of CWA. The taphonomic adjustment amplifies the oscillations pre 1,900 cal BP. However, we still observe an increase in the taphonomically adjusted mean KDE (although less pronounced) after 1,900 cal BP and then again after 1,200 cal BP. As with the Central area, the global taphonomic adjustment amplifies fluctuations of the mean KDE more and more earlier in the time-series. In addition, after 1,100 cal BP, the taphonomically adjusted KDE does not show a recession below the long-term carrying capacity (blue dashed curve) of the area. The taphonomic adjustment transforms the data to better support the expectation that the Southern region would display less of a sustained population expansion than the Northern area.

In sum, the global taphonomic adjustment amplifies fluctuations in the mean KDE

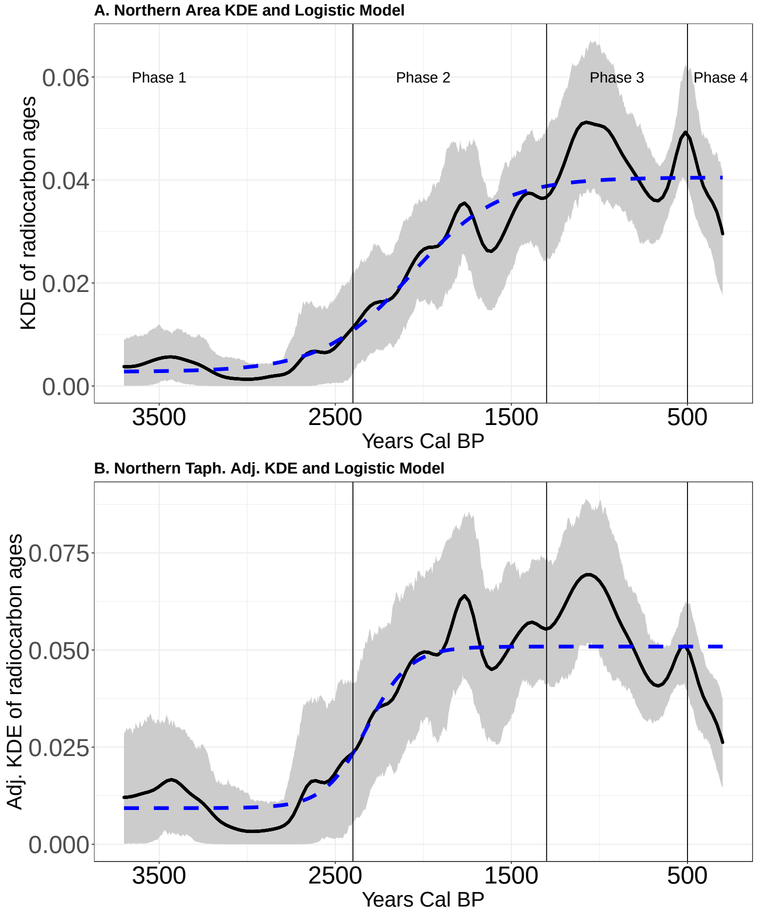

Fig 1. A-The change in mean KDE over time. Grey area is a 95 % confidence envelope. Dashed blue curve is the fit of a logistic model. B–The change in the taphohonomically adjusted mean KDE over time. Taphonomic adjustment made using the global taphonomic loss adjustment proposed by [5]. Grey area is a 95 % confidence envelope. Dashed blue curve is the fit of a logistic model.

of each area. However, the adjustment does not change the fundamental long-term structure of the mean KDEs. In the Northern area, there is a period of about 990 years of long-term expansion. In the Central and Southern sub-regions, the mean KDEs mostly oscillate around a long-term mean with only brief periods of punctuated increase. These patterns are consistent with the results presented in the main paper. In all three sub-areas, the taphonomic adjustment suggests lower mean KDEs (populations) post 1,300 cal BP than the un-adjusted mean KDEs and magnifies a trend of declines in the KDEs after 800 cal BP in the Northern and Central sub-regions. This would change our result of suspected demographic transition in the Central area after 1,300 cal BP and make the transitions after 1,300 cal BP much smaller in the Northern and Southern areas. However, we should be very cautious about interpreting the adjusted KDE as a better representation late in time.

Although the adjustment of the mean KDEs for taphonomic loss is consistent with the expectations made in the paper, we remain skeptical of the transformations. In principle, it is possible that the taphonomic process of sediment loss impacts the distributions of archaeological radiocarbon [2–4]. At this point, however, this analogical argument is better warranted for the terminal Pleistocene and early Holocene than it is

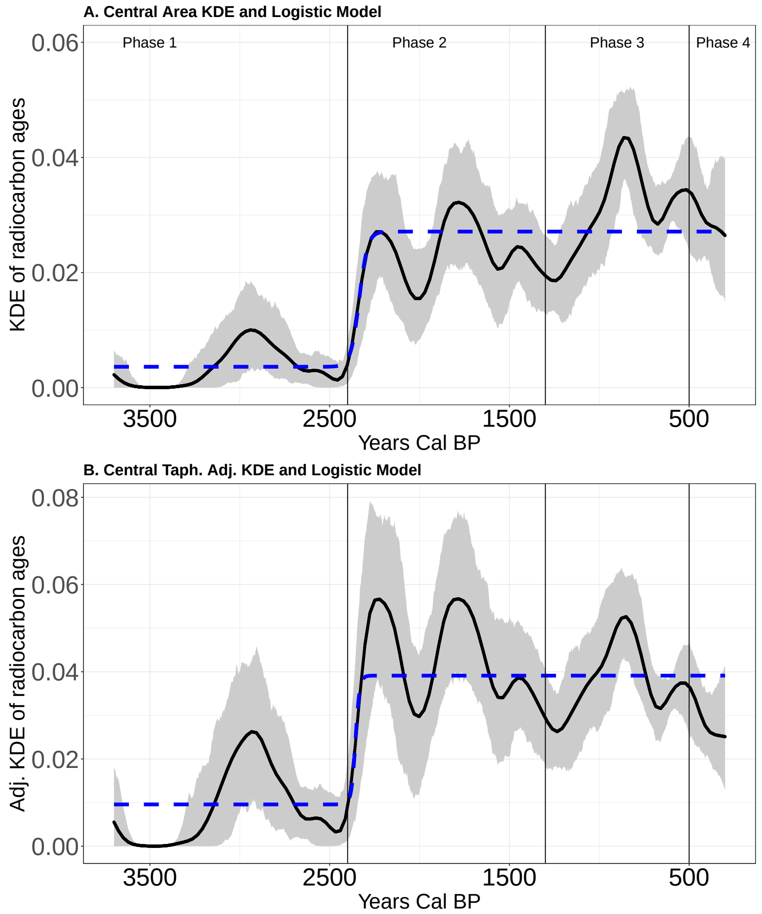

Fig 2. A-The change in mean KDE over time. Grey area is a 95% confidence envelope. Dashed blue curve is the fit of a logistic model. B–The change in the taphonomically adjusted mean KDE over time. Taphonomic adjustment made using the global taphonomic loss adjustment proposed by [5]. Grey area is a 95% confidence envelope. Dashed blue curve is the fit of a logistic model.

for the Late Holocene. There is considerable uncertainty about the values of the parameters that might describe the archaeological context loss function across geomorphic settings [6]. The sequences we analyze are drawn from large areas, which contain many kinds of geomorphic environments. Some of these environments may have experienced high rates of sediment loss, while others may preserve discarded carbon at higher or constant rates over time (e.g., many caves). More importantly, Bluhm and Surovell find that in the two global databases that they use, the uncertainty of parameter estimates increases after 6,000 cal BP and especially after 2,000 cal BP, and thus, one should be very cautious about using the adjustment after 2,000 cal BP [2]. This is one reason why researchers should have a very good *a prior* justification for applying a global taphonomic adjustment [7], especially when analyzing time-series covering the last 6,000 years.

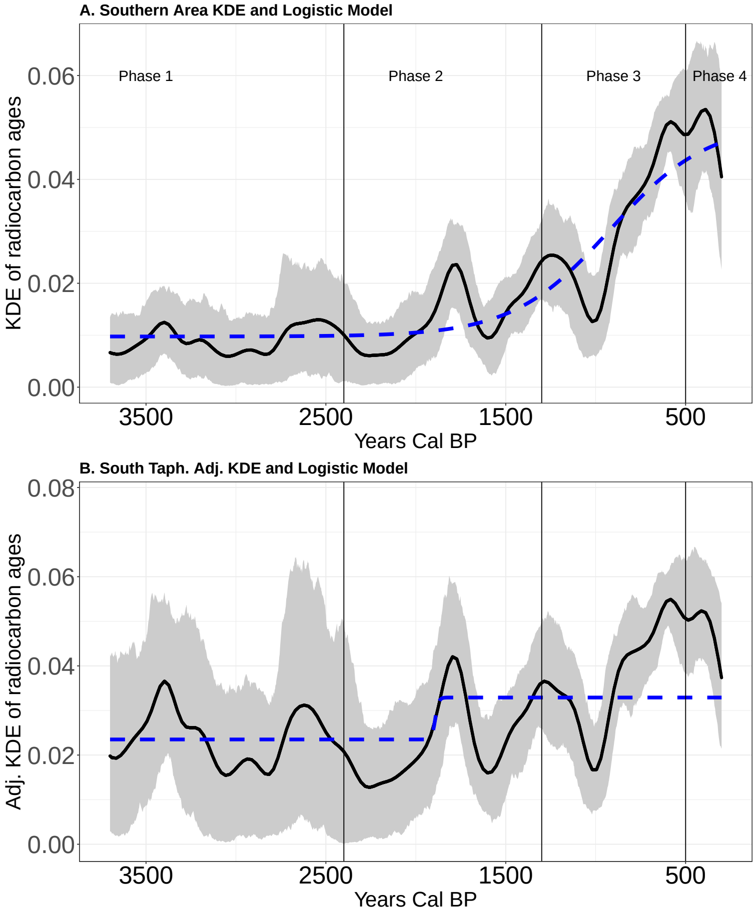

Fig 3. A-The change in mean KDE over time. Grey area is a 95 % confidence envelope. Dashed blue curve is the fit of a logistic model. B–The change in the taphohonomically adjusted mean KDE over time. Taphonomic adjustment made using the global taphonomic loss adjustment proposed by [5]. Grey area is a 95 % confidence envelope. Dashed blue curve is the fit of a logistic model.

Further Analysis of Stable Isotopes from Human Bone

In this section, we illustrate changes in human stable isotopes over time. The main result is that the isotopes suggest that people were consuming more plant resources during temporal Phases 2 and 3 in each area of CWA.

Fig. 4A-C illustrate the relationship between $\delta^{13}C$ collagen and carbonate with individuals symbolized by the phase in which they were buried. The dashed red line is the relationship between $\delta^{13}C$ collagen and carbonate holding protein source constant from C_3 feeding sources, and the green line is the same holding protein source constant from C_4 feeding sources [8]. These lines allow us to potentially infer the amount of C_3 vs. C_4 plant energy individuals consumed. Near the right endpoints of each curve, individuals consumed more C_4 plant energy and near the left endpoints, individuals consumed more C_3 plant energy. For instance, in the Central area (Figure 4A), we observe that during Phase 1 individuals cluster near the center of the C_3 protein line (red dashed line). During Phase 2, individuals display major differentiation. One cluster extends to the lower right of the C_3 protein line. This suggests a shift in diet toward some consumption of C_4 plants and consumption of C_3 protein sources. Conversely,

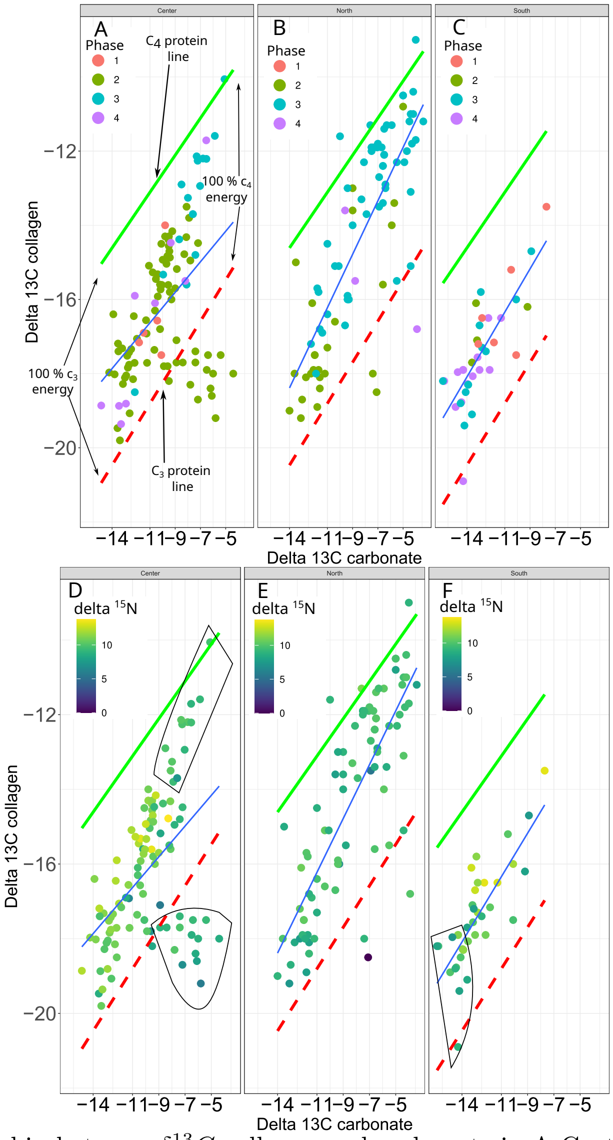

Fig 4. Relationship between $\delta^{13}C$ collagen and carbonate in A-Central, B-Northern, and C-Southern areas. In all cases, the red dashed line is a linear relationship between collagen and carbonate in controlled feeding studies among animals consuming C_3 protein, and the green line is the same relationship in controlled feeding studies among animals consuming C_4 protein. Linear models (red and green lines) calculated from controlled feeding studies redrawn from data published by [8] following [9] Dots are colored by time Phases 1-4. Graphs D-F replicate A-C, except that dots are colored by $\delta^{15}N$ isotope values. Circles indicate groups of individuals with low $\delta^{15}N$ values.

another cluster during Phase 2 spaces closer to the C_4 protein line, and another cluster overlaps with Phase 1 individuals and spaces closer to the C_3 protein line. These patterns indicate two additional groups of individuals. One group focused more on C_3 plants and protein and another group consuming more C_4 plants (maize) and protein sources. Phase 3 individuals all cluster even closer to the right endpoint of the C_4 protein line, indicating more consumption of C_4 plants (maize) and protein sources during peak population density in the region.

The North and South areas display a less complex pattern. In the North, from Phase 2 to Phase 3 individuals' diets shifted toward more consumption of C_4 plants (maize) and protein sources during peak population density in the region (Figure 4B). This is indicated by the blue dots spaced closer to the right endpoint of the C_4 protein line than the green dots. In the Southern area (Figure 4C), we observe that individuals from Phases 3 and 4 space closer to the left endpoint of the C_3 protein line than individuals from Phases 1 and 2, though the sample size is small. This suggests a shift in diet toward the consumption of more C_3 resources during the phases of peak population density in the Southern area.

Fig. 4 D-F replicates A-C, except that the individuals are now symbolized by $\delta^{15}N$ values. In the Central area, we observe two clusters of individuals potentially consuming less protein. One is the cluster of individuals during Phase 2 with a high C_4 plant diet but protein from C_3 sources. The other cluster is the group of individuals from Phase 3 who display diets higher in maize consumption. This suggests a shift toward carbohydrate consumption late in time during peak population density in the Central area. In the Northern area, $\delta^{15}N$ values are lower than the other two areas and change little through time. In the Southern area, there is a cluster of individuals with lower $\delta^{15}N$ values near the left endpoint of the C_3 protein line. At the same time, there are also individuals from Phases 3 and 4 with higher protein diets. This suggests differentiation in protein consumption, with some individuals potentially consuming more C_3 plants and others consuming more C_3 protein sources, such as camelids and armadillos.

In sum, in all three areas we observe shifts in the consumption of resources associated with increases in population density. We suggest that these shifts were toward more carbohydrate production from plants during phases of higher population density.

References

1. Neme GA, Gil AF, Garvey R, LLano CL, Zangrando A, Franchetti F, et al. El registro arqueológico de la Gruta de El Manzano y sus implicancias para la arqueología de Nordpatagonia. Magallania (Punta Arenas). 2011;39(2):243–265.

2. Bluhm LE, Surovell TA. Validation of a global model of taphonomic bias using geologic radiocarbon ages. Quaternary Research. 2019;91(1):325–328.

3. Surovell TA, Byrd Finley J, Smith GM, Brantingham PJ, Kelly R. Correcting temporal frequency distributions for taphonomic bias. Journal of Archaeological Science. 2009;36(8):1715–1724.

4. Surovell TA, Brantingham PJ. A note on the use of temporal frequency distributions in studies of prehistoric demography. Journal of Archaeological Science. 2007;34(11):1868–1877.

5. Bluhm LE, Surovell TA. Validation of a global model of taphonomic bias using geologic radiocarbon ages. Quaternary Research. 2019;91(1):325–328.

6. Ballenger JA, Mabry JB. Temporal frequency distributions of alluvium in the American Southwest: taphonomic, paleohydraulic, and demographic implications. Journal of Archaeological Science. 2011;38(6):1314–1325.

7. Williams N Alan. The use of summed radiocarbon probability distributions in archaeology: a review of methods. Journal of Archaeological Science. 2012;39(3):578 – 589.

8. Froehle AW, Kellner CM, Schoeninger MJ. FOCUS: effect of diet and protein source on carbon stable isotope ratios in collagen: follow up to Warinner and Tuross (2009). Journal of Archaeological Science. 2010;37(10):2662–2670.

9. Mauldin RP, Hard RJ, Munoz CM, Rice JL, Verostick K, Potter DR, et al. Carbon and nitrogen stable isotope analysis of hunter–gatherers from the Coleman site, a Late Prehistoric cemetery in Central Texas. Journal of Archaeological Science. 2013;40(2):1369–1381.
