# Supplementary material for: A model of long-term population growth with an application to Central West Argentina
Source: PLoS One. 2024 Aug 7;19(8):e0307703. doi: 10.1371/journal.pone.0307703 (PMC11305588; doi:10.1371/journal.pone.0307703)
Supplement: Attachment — Submitted filename: Response.pdf [file pone.0307703.s003.pdf]

Utah State University
Dr. Jacob Freeman, Associate Professor

Academic Editor
PLoS One

May 28, 2024

Dear Dr. Iovita,

I am writing on behalf of my coauthors to re-submit an original research article titled: “A model of long-term population growth with an application to Central West Argentina." Thank you for the opportunity to respond to the Reviewers’ comments and improve the manuscript. Following the advice of the Reviewers, we have made three substantive changes to improve the manuscript and bring the work in-line with Plos One publication criteria. First, we revised the Data and methods section and added a new supporting document (S1 Appendix) to discuss how taphonomy might impact our results. Specifically, we illustrate that changes in occupation intensity are evidenced beyond the archaeological radiocarbon record in Central West Argentina, and we present the impact of using a global taphonomic correction on the structure of the KDEs used in the main analysis. Second, we revised the R-code and posted the code and data sets as requested by Reviewer #1 (please see Freeman (2024)). Third, we reorganized the paper, moving detailed isotope analyses to the S1 Appendix, and we have attempted to clarify the contribution of the paper. Please find appended to this letter a detailed response to reviewers, a revised manuscript, and a revised S1 Appendix.

Thank you in advance for considering our manuscript for publication.

Sincerely Yours,

Jacob Freeman
Associate Professor
Anthropology Program
The Ecology Center
Utah State University
Jacob.Freeman@usu.edu

Specific Responses to Reviewers

Editor Comments:

1. Please ensure that your manuscript meets PLOS ONE's style requirements, including those for file naming. Done.

2. We note that the grant information you provided in the 'Funding Information' and 'Financial Disclosure' sections do not match.

When you resubmit, please ensure that you provide the correct grant numbers for the awards you received for your study in the 'Funding Information' section.

3. Thank you for stating the following financial disclosure: "We are thankful for the support of research grant PICT 2021-I-A-00891"

Please state what role the funders took in the study. If the funders had no role, please state: ""The funders had no role in study design, data collection and analysis, decision to publish, or preparation of the manuscript."" If this statement is not correct you must amend it as needed. Please include this amended Role of Funder statement in your cover letter; we will change the online submission form on your behalf.

The funders had no role in study design, data collection and analysis, decision to publish, or preparation of the manuscript.

4. Thank you for stating the following in the Acknowledgments Section of your manuscript: "We are grateful for support for Past Global Changes (PAGES) project, which in turn received support from the Swiss Academy of Sciences and the Chinese Academy of Sciences. We are greatful for the support of research grant PICT 2021-I-A-00891, and to the Museo Moyan, Museo FFyL Canals Frau, Museo de Historia Natural de San Rafaeal-Humberto Lagiglia, and Museo Regjonal de Malargue-Jorge Luna for support accessing samples and data."

We note that you have provided funding information that is not currently declared in your Funding Statement. However, funding information should not appear in the Acknowledgments section or other areas of your manuscript. We will only publish funding information present in the Funding Statement section of the online submission form. Please remove any funding-related text from the manuscript and let us know how you would like to update your Funding Statement. Currently, your Funding Statement reads as follows: "We are thankful for the support of research grant PICT 2021-I-A-00891"

Please include your amended statements within your cover letter; we will change the online submission form on your behalf.

Amended funding statement: This research was supported by the Agencia Nacional de Promoción de la Investigación, el Desarrollo Tecnológico y la Innovación [PICT 2019–04447; PICT 2020-0684, PICT 2021–I-A-00891, and PICT 2022-04-00016], the Consejo Nacional de Investigaciones Científicas y Técnicas, and the Universidad Tecnológica Nacional. We are also thankful for support from the Past Global Changes (PAGES) project, which in turn received support from the Swiss Academy of Sciences and the Chinese Academy of Sciences. .

5. We note that Figure 4 in your submission contain map images which may be copyrighted. All PLOS content is published under the Creative Commons Attribution License (CC BY 4.0), which means that the manuscript, images, and Supporting Information files will be freely available online, and any third party is permitted to access, download, copy, distribute, and use these materials in any way, even commercially, with proper attribution. For these reasons, we cannot publish previously copyrighted maps or satellite images created using proprietary data, such as Google software (Google Maps, Street View, and Earth)

According to PLoS ONE:

Esri's use of open source software libraries is disclosed in the Third-Party OSS-FOSS Acknowledgement Documents found at the link below.

Open Source Acknowledgements (https://links.esri.com/open-source-acknowledgments)

ArcGIS Notebooks makes use of The FreeType Project, following The FreeType Project License (https://git.savannah.gnu.org/cgit/freetype/freetype2.git/tree/docs/FTL.TXT).

We have added the following line to the Fig. 4 caption:

"Copyright 1995-2024 ESRI. All rights reserved. Published in the United States of America."

Reviewer #1:

In this article, the authors present an analysis of summed probability distributions of radiocarbon dates from Central Western Argentina (CWA) to demonstrate multiple population expansions and regressions during the Holocene in this region. These demographic changes are then interpreted in the context of the Ideal Specialization Model to argue for successive population expansions that are associated with multiple innovations in food production instead of a single agricultural demographic transition.

While this paper does attempt to make good use of available data to answer questions about demographic trends in the Holocene record of CWA, there are major issues with this paper including: 1) lack of taphonomic correction for radiocarbon dates and 2) lack of concrete archaeological data for contextualizing the authors' claims. For these reasons, I would suggest major revisions for this article.

Thank you for reviewing the manuscript and providing constructive critiques.

While this paper does attempt to make good use of available data to answer questions about demographic trends in the Holocene record of CWA, there is a major issue with this paper – the lack of taphonomic correction for the radiocarbon dates. The authors state that they do not use any taphonomic adjustments because those can introduce "complexity" and "uncertainity." I do not think this justification is sufficient, especially since the only citation they offer as proof is their own work that is still under review. Sure, incorporating taphonomic adjustments may introduce some bias, particularly if the global taphonomic correction is used; however, the authors' data is heavily skewed towards later dates as anyone can see from creating a quick histogram of their provided data. Furthermore, the jump in the number of radiocarbon dates corresponds to around 2500 calBP, which is exactly when the authors argue for their demographic transitions. Why should we be convinced this is a true demographic trend if taphonomy, which has been shown to affect the interpretation of demography from radiocarbon dates (see Surovell's work, some of which the authors cite), is not considered? Could the authors not construct their own taphonomic correction function sensu Surovell et al 2009 based on data from the CWA? At least show us what the differences would be between the curves produced with and without a taphonomic correction (global or otherwise). For this reason, I would suggest major revisions for this article.

In this revised version of the paper, we discuss the potential that the radiocarbon data increase at 2500 cal BP in the Central area, 2950 cal BP in the North, and ca. 1900 cal BP in the South are related to changes in the preservation of archaeological contexts over time. We also explore the impact of the global taphonomic adjustment and the limitations of this approach. Specifically, we revise the Data and Methods section to include a more detailed discussion of taphonomy. Further, we have created a S1 Appendix. In this appendix we (1) discuss the effects of using a global taphonomic adjustment on our main results , and (2) cite evidence from stratified cave deposits that indicate occupation intensity was lowest in the region between 7,000 and 3,500 cal BP and then increased after 3,000 and especially after 2,000 cal BP.

In addition to the issue of taphonomic bias, the overall organization of the paper makes it very difficult to follow the authors' points and the presentation of key archaeological data is missing, leaving the reader confused by some of the arguments the authors make. I detail these points below in order of appearance in the paper.

1) Description of Ideal Specialization Model (ISM) The authors should summarize the key points more concisely to get to the meat of the paper more quickly. For example, lines 41-63 explain findings from a different paper by the same first

author to justify including climate variability in the ISM. These paragraphs could be shortened to simply say in this paper the ISM is extended to include climate variability and then immediately dive into the equation derivation. Similarly, lines 155-166 are summarizing points that are made again in the Model Dynamics subsections, rendering this initial summary unnecessary. The authors should thoroughly reread the Model Dynamics section to make their points more succinct.

Additionally, throughout this section (and the whole paper, really), authors should clarify their terminology and variables. For example, the ISM seems to hinge on a measure of per capita fitness. The authors should specify that this value is tracking the population growth rate. For those less familiar with demographic research, the equivalence between per capita fitness, per capita growth rate, and population growth rate is unclear. Additionally, the variables d and fmin are not explicitly defined as would be typically when presenting equations. Furthermore, in the Model Dynamics sections the authors need to standardize the terms they are using. Many terms are used interchangeably in text and figures (e.g. per capita fitness = population growth, population size = population density, etc).

In the revised manuscript, we follow the Reviewer's advice by 1) reducing redundant text, and 2) making sure that all terms are defined at first use and used consistently throughout the manuscript.

Specifically:

1) "For example, lines 41-63 explain findings from a different paper by the same first author to justify including climate variability in the ISM. These paragraphs could be shortened to simply say in this paper the ISM is extended to include climate variability and then immediately dive into the equation derivation."

We have deleted these paragraphs and moved the most relevant information to the Introduction.

2) "Similarly, lines 155-166 are summarizing points that are made again in the Model Dynamics subsections, rendering this initial summary unnecessary."

We respectfully disagree with the reviewer on this point. The summary here allows a reader to quickly grasp the main points of the section and then either continue reading the subsections for the details or move to the next section.

3) " For example, the ISM seems to hinge on a measure of per capita fitness. The authors should specify that this value is tracking the population growth rate. For those less familiar with demographic research, the equivalence between per capita fitness, per capita growth rate, and population growth rate is unclear"

In the revised manuscript, we clarify that the per capita growth rate and per capita fitness are equivalent. The equivalence is defined in equation 2 ($f \equiv \dot{p}/p$), where the per capita fitness of a population is the growth rate $\dot{p}$ divided by population size, p (i.e., *per capita* population growth).

We are unsure of the Reviewer's intended meaning by "the ISM seems to hinge on a measure of per cpaita fitness." The logistic model and its many formulations capture the basic assumption that individual reproduction is density-dependent. This means that as population size within a fixed area increases *per capita* fitness declines as defined in equation 2. This is an outcome of the assumption made in all models with a Malthusian component that competition for resources limits the ability of individuals to convert the resources of an area into successful reproduction.

2) Presentation of archaeological data and methods The section on the timing of the onset of agriculture and food production/collection technologies in the CWA needs to be expanded, especially considering the authors are concerned with associating radiocarbon dates with the onset of these innovations. The authors should provide a table of dates and descriptions for the innovations they reference for each subregion (these dates could also be incorporated as vertical lines in their results figures).

We now include a table noting the timing of changes in subsistence, settlement, and other technology as currently known. In addition, we have re-written this section to better articulate that the Temporal Phases 1-4 mark major changes in technology and adaptations in general in CWA. Please keep in mind that this region of

the world has much less archaeological research conducted than, say, is typical in N. America or Europe.

3) Presentation of results For the section on population expansion and resources, the authors document in detail the different demographic trends and how those relate to the diet changes (isotope data) and innovations (which as stated above need to be better evidenced with dates). However, some aspects of the results are a bit difficult to follow due to organization. First, there is a lot of comparison between the KDE curves between the different subregions; is it possible to plot the curves for each subregion on the same graph so the reader can visualize the differences the authors point out? Second, is there a methodology behind the selection of the sections outlined in the blue boxes for the per capita growth curves? Different types of trends seem to be highlighted between the different subregions.

For the isotope data, I understand why the authors included data to characterized different aspects of diet. However, the section presenting the isotope results is long and confusing. The authors are trying to describe all the nuances in the data, when it would be clearer if they summarized it (e.g. shifts to high C4, low protein diets in Phase 3 for the North and Central region, indicating perhaps a focus on agriculturally derived food sources).

(1) In the revised manuscript, we reorganize the results section around three expectations suggested by the ISM model. We added Fig. 5 to compare the population dynamics of the three regions of CWA.

(2) In the revised manuscript, we move the detailed discussion of the isotopes to the S1 Appendix.

Finally, the authors seem to be very variable in their treatment of the Southern subregion. In some instances, it is lumped in with the North and Central subregions as also undergoing a shift to a plant focused diet, but there is clearly a different pattern in the south with an increased focus on C3 plants versus the C4 trend seen in the other two regions. Why is the southern region so different? Can it be solely attributed to the dominance of hunter-gatherers throughout the study period? The authors seem to suggest there is some sort of innovation or intensification of food production related to wild plants in the Southern region but never really elaborate on what this means. Is there archaeological evidence of cultivation of C3 plants?

We attempt to make clear that we suspect that hunter-gatherers intensified their resource production in the Southern area. There is evidence of the increased use of mesquite pods and larger grinding stones as noted in the revised Table 1.

4) Discussion and conclusions When discussing the mechanisms behind population change in the CWA, the authors seem to make the argument that they must be associated with innovation and not climate. This seems at odds with the initial presentation of the ISM which was specifically modified to include climate variability. Is there climate variability during the study period in the CWA? In the Model Dynamics different climate suitability seems to affect the size and timing of demographic transitions, but climate is not discussed in subsequent sections beyond to say the various demographic transitions could not be explained by climate alone. Is it possible that the prolonged population expansion in the North is due to better climatic conditions there?

Thank you for pointing out details related to climate. We now see that we were not clear enough in our description of climate effects on population. In the revised manuscript, we clarify that there is a difference between the long-term, climate driven suitability of a habitat and shorter-term fluctuations in climate that can act as a mechanism for innovation. The fist process is a constraint on long-run population density; the second process is a mechanism that might keep a population from getting stuck in a Malthusian trap. We also now raise the possibility that the North was a better suited environment for maize production in general than the Central and Southern areas.

Some of the statements made in the discussion and conclusion sections need to be contextualized by the limit of this study to just the CWA. For example, lines 719-726 make some broad statements about the effects of innovation on population changes. Are the authors confident that these statements apply to every situation of demographic transitions? If not, this should be contextualized as a possible scenario based on the evidence from the CWA.

In the revised manuscript, we attempt to contextualize statements about processes operating in general and make clear where we are speculating.

Overall, I think this paper could offer an interesting case study for studying how demographic transitions occur differently in areas in close proximity. However, in its current state, the paper cannot offer any convincing insights into the suite of mechanisms behind demographic trends without providing us with: 1) better justification for not including taphonomic bias (or better yet, redoing the analyses with taphonomic correction), 2) more concrete information on the archaeological context and how that relates to the differences in demographic trends between the subregions, and 2) more information on how climatic variability fits into this story. If the authors can make these changes, then this paper stands to make a good contribution to discussions of the diversity of effects that food production intensification had on demography at both a local and global scale.

Minor comments:

Need to review the figure labels throughout the paper. There are figures that are referenced that are missing (e.g. Figure 2E and 2F). There also seem to be written comparisons between two different figures that reference the same figure number (e.g. lines 463-465) fixed.

A few citations are incorrectly formatted

fixed.

Position of figure 8 does not correspond with its reference in the text

We attempted to fix.

The authors frequently use generations as a time unit even though all the graphs are given in years calBP. It would be helpful to perhaps include generation calculations as a secondary X-axis or to at least tell the reader how many years are included in a generation by the authors' definition

we now include the definition of a generation as 30 years.

In the section presented the results from the causal impact analysis, the second analysis of the post 2500 calBP period is not well-explained. Is this analysis being used to look at whether or not there were subsequent innovations to explain changes in population following the initial population expansion around 2500 calBP?

Yes. The revised manuscript explains this in more detail. The revised Fig. 5 helps illustrate the potential demographic expansions in each region and the causal impact analysis attempts to understand if the long-term climate trend alone can explain the demographic expansions.

Code review:

I appreciate the authors providing the code used in their analyses and for providing comments throughout documenting different analysis steps. However, in its current state it does not run completely, so I cannot fully evaluate it. With the provided data, the R script runs until line 54, when table "Updated/North30Sum.csv" is supposed to be loaded. Presumably, this table is the above table written to CSV in line 48, but those tables do not have the same name in the script. Even changing the name produces an error because the column names are not correct.

We have revised the code and posted on github with a persistent identifier in Zenodo Freeman (2024). We have attempted to address all of the points below.

The comment starting at line 100 describes what the authors do to calculate two columns outside of the R environment. These steps can be accomplished in R using the apply()and the dplyr::lead() functions; this should be implemented in R to make the analysis entirely reproducible from the script alone.

Even when making my own columns for MKDE and PerCapita, I cannot reproduce the graph from line 153 – missing columns "lo" and "hi"

For graph produced on line 178, again missing a column "PerCap" – this needs to be created in R

For both the South and Central region sections of the code – errors produced when trying to run the binPrep() function. Not being able to run these sections makes it impossible to run anything in the Causal Impact analysis section.

In the Isotopes sections – need to install/load package for conover.test()

Finally, it does not seem like the script produces all of the figures for the results of each subregion (Figure 5-7). Only the code for Figure 5, southern region is included.

Reviewer #2

While I am not familiar with the simulation models the authors use, I am aware of the data on which they are based and their previous publications in which they have been discussed. Therefore I consider that this work fits perfectly with the Plos One criteria.

The manuscript seems to me to be an excellent contribution to the understanding of the topic discussed about the population pulses in different sectors of the Central West of Argentina, in relation to the total or partial adoption of corn within their diet. My only objection is that there is a need to delve into socio-political aspects related to the interaction between different populations of the macro region of study that may have influenced changes in the subsistence patterns analyzed.

Thank you for reviewing the paper. We agree that interactions among the populations were important, and we hope to develop this line of research more in the future. We have revised the discussion to note this possibility.

References

Freeman, J. (2024). people3k/cwa_ism_data: CWA_ISM_DATA, deposited May 30, 2024: 10.5281/zenodo.11390810.
