# Supplementary material for: A model of long-term population growth with an application to Central West Argentina
Source: PLoS One. 2024 Aug 7;19(8):e0307703. doi: 10.1371/journal.pone.0307703 (PMC11305588; doi:10.1371/journal.pone.0307703)
Supplement: Attachment — Submitted filename: Response.pdf [file pone.0307703.s004.pdf]

Utah State University
Dr. Jacob Freeman, Associate Professor

Academic Editor
PLoS One

July 5, 2024

Dear Dr. Iovita,

I am writing on behalf of my coauthors to re-submit an original research article titled: “A model of long-term population growth with an application to Central West Argentina." Thank you for the opportunity to improve the manuscript. Following the advice of the Reviewer, we have made minor changes to clarify the Model Dynamics section of the paper. Please find appended to this letter a detailed response to the Reviewer, a revised manuscript, and a revised S1 Appendix.

Thank you in advance for considering our manuscript for publication.

Sincerely Yours,

Jacob Freeman
Associate Professor
Anthropology Program
The Ecology Center
Utah State University
Jacob.Freeman@usu.edu

Specific Responses to Reviewers

Reviewer #1:

Thank you for reviewing the manuscript and providing constructive critiques.

1) In Figure 1, the numbered curves are confusing because they seem to suggest 4 infrastructure systems despite the authors saying they assume a ceiling of A2. Does the occurrence of an innovation necessarily push a population into a new infrastructure system or does the model allow for multiple innovations to occur in A1 and A2?

In the revised manuscript we clarify that Fig. 1 illustrates how population would expand in the absence of an innovation ceiling (Fig. 1b) and in the absence of a climatic ceiling (i.e., the mean climate can only improve so much (Fig. 1a).

Please see lines 145-154: "We present the results of three sets of numerical experiments in the paper. In the first set of experiments, we study a simple version of the ISM to clearly define the mechanisms of long-term population expansion. First, we simplify the ISM by treating mean climate as the only parameter that determines $K_i(t)$ (i.e., innovation is impossible and population density has no effect on $K_i(t)$). Second, we hold climate constant, relax the assumption of an innovation ceiling, and assume that A_i is the only parameter that impacts $K_i(t)$. In the second and third sets of experiments, we progressively increase the complexity of the model to understand how the presence of an innovation ceiling, the feedback of human population density on the abundance of resources, $K_i(t)$, and short-term climate variation impact population dynamics." This strategy of progressively adding complexity to a model is a common tactic to study dynamic models to clearly understand how the interaction of parameters impacts the dynamic variable.

2) It is only in the caption of Figure 2 that it is explained that $A = A2 - A1$. This should be moved to the main text (perhaps on line 248) to make it clearer that $A = 3$ does not mean A3 exists.

In the revised manuscript we follow the Reviewer's advice and move this to the main text.

3) It is extremely difficult to see the black curve in Figure 3 with the overlaid green and red lines. This makes it very hard for the reader to comprehend the effects of climate variability to keep the population from reaching equilibrium or to force oscillation between infrastructure systems (i.e. the patterns that authors describe in lines 293-295). Is there a better way to visualize this, especially since the green curve is not meant to be a central tendency of the black curve but a separate pattern altogether?

The effect of climate variability is to induce a demographic transition (i.e., keep the system from reaching equilibrium). The fast oscillation between infrastructure systems signals the coming demographic transition. In the revised manuscript, we dash the colored lines and annotate the graphs to better highlight the patterns.

4) There is a spelling error in Figure 1 – should be "population density" fixed.

5) In figure 1 caption, should "s" be capitalized to match S in the previous equations?

Yes. Fixed.

6) In figure 3b, is it possible to color the lines to match the green used in figure 3a that is showing what will occur without climate variability?

In the revised manuscript, we use red to illustrate what happens with climate variability. Without climate variability, the system simply enters equilibrium. With climate variability, the system experiences a demographic transition.

References
