# Supplementary material for: A model of long-term population growth with an application to Central West Argentina
Source: PLoS One. 2024 Aug 7;19(8):e0307703. doi: 10.1371/journal.pone.0307703 (PMC11305588; doi:10.1371/journal.pone.0307703)
Supplement: Attachment — Submitted filename: PONE-D-24-04698_review..docx [file pone.0307703.s002.docx]

In this article, the authors present an analysis of summed probability distributions of radiocarbon dates from Central Western Argentina (CWA) to demonstrate multiple population expansions and regressions during the Holocene in this region. These demographic changes are then interpreted in the context of the Ideal Specialization Model to argue for successive population expansions that are associated with multiple innovations in food production instead of a single agricultural demographic transition.

While this paper does attempt to make good use of available data to answer questions about demographic trends in the Holocene record of CWA, there is a major issue with this paper – the lack of taphonomic correction for the radiocarbon dates. The authors state that they do not use any taphonomic adjustments because those can introduce “complexity” and “uncertainity.” I do not think this justification is sufficient, especially since the only citation they offer as proof is their own work that is still under review. Sure, incorporating taphonomic adjustments may introduce some bias, particularly if the global taphonomic correction is used; however, the authors’ data is heavily skewed towards later dates as anyone can see from creating a quick histogram of their provided data. Furthermore, the jump in the number of radiocarbon dates corresponds to around 2500 calBP, which is exactly when the authors argue for their demographic transitions. Why should we be convinced this is a true demographic trend if taphonomy, which has been shown to affect the interpretation of demography from radiocarbon dates (see Surovell’s work, some of which the authors cite), is not considered? Could the authors not construct their own taphonomic correction function sensu Surovell et al 2009 based on data from the CWA? At least show us what the differences would be between the curves produced with and without a taphonomic correction (global or otherwise). For this reason, I would suggest major revisions for this article.

In addition to the issue of taphonomic bias, the overall organization of the paper makes it very difficult to follow the authors’ points and the presentation of key archaeological data is missing, leaving the reader confused by some of the arguments the authors make. I detail these points below in order of appearance in the paper.

*1) Description of Ideal Specialization Model (ISM)*

The authors should summarize the key points more concisely to get to the meat of the paper more quickly. For example, lines 41-63 explain findings from a different paper by the same first author to justify including climate variability in the ISM. These paragraphs could be shortened to simply say in this paper the ISM is extended to include climate variability and then immediately dive into the equation derivation. Similarly, lines 155-166 are summarizing points that are made again in the Model Dynamics subsections, rendering this initial summary unnecessary. The authors should thoroughly reread the Model Dynamics section to make their points more succinct.

Additionally, throughout this section (and the whole paper, really), authors should clarify their terminology and variables. For example, the ISM seems to hinge on a measure of per capita fitness. The authors should specify that this value is tracking the population growth rate. For those less familiar with demographic research, the equivalence between per capita fitness, per capita growth rate, and population growth rate is unclear. Additionally, the variables *d* and *fmin* are not explicitly defined as would be typically when presenting equations. Furthermore, in the Model Dynamics sections the authors need to standardize the terms they are using. Many terms are used interchangeably in text and figures (e.g. per capita fitness = population growth, population size = population density, etc).

*2) Presentation of archaeological data and methods*

The section on the timing of the onset of agriculture and food production/collection technologies in the CWA needs to be expanded, especially considering the authors are concerned with associating radiocarbon dates with the onset of these innovations. The authors should provide a table of dates and descriptions for the innovations they reference for each subregion (these dates could also be incorporated as vertical lines in their results figures).

The authors should review (see code review below) their R script to make sure it can run with just the two datasets provided.

*3) Presentation of results*

For the section on population expansion and resources, the authors document in detail the different demographic trends and how those relate to the diet changes (isotope data) and innovations (which as stated above need to be better evidenced with dates). However, some aspects of the results are a bit difficult to follow due to organization. First, there is a lot of comparison between the KDE curves between the different subregions; is it possible to plot the curves for each subregion on the same graph so the reader can visualize the differences the authors point out? Second, is there a methodology behind the selection of the sections outlined in the blue boxes for the per capita growth curves? Different types of trends seem to be highlighted between the different subregions.

For the isotope data, I understand why the authors included data to characterized different aspects of diet. However, the section presenting the isotope results is long and confusing. The authors are trying to describe all the nuances in the data, when it would be clearer if they summarized it (e.g. shifts to high C4, low protein diets in Phase 3 for the North and Central region, indicating perhaps a focus on agriculturally derived food sources).

Finally, the authors seem to be very variable in their treatment of the Southern subregion. In some instances, it is lumped in with the North and Central subregions as also undergoing a shift to a plant focused diet, but there is clearly a different pattern in the south with an increased focus on C3 plants versus the C4 trend seen in the other two regions. Why is the southern region so different? Can it be solely attributed to the dominance of hunter-gatherers throughout the study period? The authors seem to suggest there is some sort of innovation or intensification of food production related to wild plants in the Southern region but never really elaborate on what this means. Is there archaeological evidence of cultivation of C3 plants?

*4) Discussion and conclusions*

When discussing the mechanisms behind population change in the CWA, the authors seem to make the argument that they must be associated with innovation and not climate. This seems at odds with the initial presentation of the ISM which was specifically modified to include climate variability. Is there climate variability during the study period in the CWA? In the Model Dynamics different climate suitability seems to affect the size and timing of demographic transitions, but climate is not discussed in subsequent sections beyond to say the various demographic transitions could not be explained by climate alone. Is it possible that the prolonged population expansion in the North is due to better climatic conditions there?

Some of the statements made in the discussion and conclusion sections need to be contextualized by the limit of this study to just the CWA. For example, lines 719-726 make some broad statements about the effects of innovation on population changes. Are the authors confident that these statements apply to every situation of demographic transitions? If not, this should be contextualized as a possible scenario based on the evidence from the CWA.

Overall, I think this paper could offer an interesting case study for studying how demographic transitions occur differently in areas in close proximity. However, in its current state, the paper cannot offer any convincing insights into the suite of mechanisms behind demographic trends without providing us with: 1) better justification for not including taphonomic bias (or better yet, redoing the analyses with taphonomic correction), 2) more concrete information on the archaeological context and how that relates to the differences in demographic trends between the subregions, and 2) more information on how climatic variability fits into this story. If the authors can make these changes, then this paper stands to make a good contribution to discussions of the diversity of effects that food production intensification had on demography at both a local and global scale.

Minor comments:

- Need to review the figure labels throughout the paper. There are figures that are referenced that are missing (e.g. Figure 2E and 2F). There also seem to be written comparisons between two different figures that reference the same figure number (e.g. lines 463-465)
- A few citations are incorrectly formatted
- Position of figure 8 does not correspond with its reference in the text
- The authors frequently use generations as a time unit even though all the graphs are given in years calBP. It would be helpful to perhaps include generation calculations as a secondary X-axis or to at least tell the reader how many years are included in a generation by the authors’ definition
- In the section presented the results from the causal impact analysis, the second analysis of the post 2500 calBP period is not well-explained. Is this analysis being used to look at whether or not there were subsequent innovations to explain changes in population following the initial population expansion around 2500 calBP?

*Code review –*

I appreciate the authors providing the code used in their analyses and for providing comments throughout documenting different analysis steps. However, in its current state it does not run completely, so I cannot fully evaluate it. With the provided data, the R script runs until line 54, when table “Updated/North30Sum.csv” is supposed to be loaded. Presumably, this table is the above table written to CSV in line 48, but those tables do not have the same name in the script. Even changing the name produces an error because the column names are not correct.

The comment starting at line 100 describes what the authors do to calculate two columns outside of the R environment. These steps can be accomplished in R using the apply()and the dplyr::lead() functions; this should be implemented in R to make the analysis entirely reproducible from the script alone.

Even when making my own columns for MKDE and PerCapita, I cannot reproduce the graph from line 153 – missing columns “lo” and “hi”

For graph produced on line 178, again missing a column “PerCap” – this needs to be created in R

For both the South and Central region sections of the code – errors produced when trying to run the binPrep() function. Not being able to run these sections makes it impossible to run anything in the Causal Impact analysis section.

In the Isotopes sections – need to install/load package for conover.test()

Finally, it does not seem like the script produces all of the figures for the results of each subregion (Figure 5-7). Only the code for Figure 5, southern region is included.
